# Uncertainty and sensitivity analyses of extinction probabilities suggest that adult female mortality is the weakest link for populations of tsetse (*Glossina* spp)

**DOI:** 10.1371/journal.pntd.0007854

**Published:** 2020-05-11

**Authors:** Elisha B. Are, John W. Hargrove

**Affiliations:** Centre of Excellence in Epidemiological Modelling and Analysis (SACEMA), University of Stellenbosch, Stellenbosch, South Africa; Kenya Agricultural and Livestock Research Organization, KENYA

## Abstract

**Background:**

A relatively simple life history allows us to derive an expression for the extinction probability of populations of tsetse, vectors of African sleeping sickness. We present the uncertainty and sensitivity analysis of the extinction probability, to offer key insights into factors affecting the control or eradication of tsetse populations.

**Methods:**

We represent tsetse population growth as a branching process, and derive closed form estimates of population extinction from that model. Statistical and mathematical techniques are used to analyse the uncertainties in estimating extinction probability, and the sensitivity of the extinction probability to changes in input parameters representing the natural life history and vital dynamics of tsetse populations.

**Results:**

For fixed values of input parameters, the sensitivity of extinction probability depends on the baseline parameter values. Extinction probability is most sensitive to the probability that a female is inseminated by a fertile male when daily pupal mortality is low, whereas the extinction probability is most sensitive to daily mortality rate for adult females when daily pupal mortality, and extinction probabilities, are high. Global uncertainty and sensitivity analysis show that daily mortality rate for adult females has the highest impact on the extinction probability.

**Conclusions:**

The high correlation between extinction probability and daily female adult mortality gives a strong argument that control techniques which increase daily female adult mortality may be the single most effective means of ensuring eradication of tsetse population.

## Author summary

Tsetse flies (*Glossina* spp.) are vectors of African trypanosomiasis, a deadly disease commonly called sleeping sickness in humans and nagana in livestock. The relatively simple life history of tsetse enabled us to model its population growth as a stochastic branching process. We derived a closed-form expression for the probability that a population of tsetse goes extinct, as a function of death, birth, development and insemination rates in female tsetse. We analyzed the sensitivity of the extinction probability to the different input parameters, in a bid to identify parameters with the highest impact on extinction probability. This information can, potentially, inform policy direction for tsetse control/elimination. In all the scenarios we considered for the global sensitivity analysis, the daily mortality rate for adult females had the greatest impact on the magnitude of extinction probability. Our findings suggest that the mortality rate in the adult females is the weakest link in tsetse life history, and this fact should be exploited in achieving tsetse population control, or even eradication.

## Introduction

Tsetse flies (*Glossina* spp.) are biting flies of both public health and economic importance in many Sub-Saharan African countries. They feed exclusively on the blood of vertebrates—game animals and livestock, and also humans, and provide the link that drives the transmission of African trypanosomiasis, a tropical disease caused by protozoan parasites of the genus *Trypanosoma*. The disease is called sleeping sickness in humans and is caused by two sub-species of *T. brucei*. In livestock the disease is termed nagana and is caused primarily by *T. vivax* and *T. congolense*. According to a WHO 2018 factsheet for human sleeping sickness, the disease still occurs in about 36 countries in Africa, mostly among poor farmers living in rural areas. Due to sustained disease and vector control efforts, the number of cases of the sleeping sickness has declined substantially in recent years. In 2015 there were about 2804 cases recorded: 97% of these were chronic infections with *T. brucei gambiense* [1]. To sustain the reduction in cases, it is important to continue to improve understanding of the tsetse fly vector, in a bid to develop more effective control techniques, with improved cost effectiveness, for the control of trypanosomiasis—whether directly through the use of trypanocides, or indirectly through reducing tsetse fly numbers.

A recent study [2] employed the theory of branching processes to derive an expression for the extinction probability for closed populations of tsetse. This equation involves numerous parameters representing death, development and fertility rates during the fly’s lifecycle. The study made suggestions regarding the parameters of prime importance in affecting the probability of extinction. The principal aim of the present study is to take the analysis further, carrying out formal uncertainty and sensitivity analyses of all of the parameters involved in the model of population growth. Sensitivity analysis is often used to investigate the robustness of model output to parameter values [3–5], but has not yet been applied to the factors affecting extinction probabilities of tsetse population.

In order to carry out these analyses, we use the branching process model developed by Kajunguri et al [2] and Hargrove [6] for the reproductive performance of female tsetse flies inseminated by a fertile male. We then use a framework, developed by Harris [7], to derive a fixed point equation for the extinction probability for a tsetse population. This approach allows us to obtain the same expression for extinction probability as [2], but it is derived with fewer steps and with less mathematical complexity. We carry out local sensitivity analysis of the extinction probability, with respect to all input parameters, at two fixed baseline values of those parameters. To identify the most important input parameters, we then use Latin Hypercube Sampling (LHS) and Partial Rank Correlation Coefficient (PRCC) methods for global uncertainty and sensitivity analyses of the extinction probability. LHS was first applied in epidemiological modelling by Blower [9, 10]. Several studies have since applied LHS in disease modelling, detailing its advantage over other sampling methods and describing the methodology concisely [8–11]. PRCC has been used widely in determining the sensitivity of models of various systems [8], [12, 13] especially to assess the sensitivity of disease models to various input parameters. Combining LHS and PRCC provides a robust method for assessing the uncertainty and the sensitivity of the extinction probability to all input parameters. Finally, we discuss what insights the results provide for policy makers considering the control, or eradication, of tsetse and trypanosomiasis.

## Materials and methods

Here we develop a stochastic model for tsetse population growth in the form of a branching process and use the model to obtain a fixed point equation for the extinction probability of tsetse populations [2, 6, 14]. We develop the branching process focusing only on female tsetse flies [6]. We follow a framework developed in [6], assuming a female tsetse fly is fertilized with probability *ϵ* and survives to deposit her first larva with probability λ^*ν*+*τ*^: where *ν* is days to first ovulation, *τ* is the inter-larval period, and λ is the adult female daily survival probability. The pupa she produces is female with probability *β*, and survives to adulthood with probability *ϕ*^*g*^ (where *g* is the pupal duration and *ϕ* is the daily survival probability of the pupa). The mother dies before the next pregnancy, having produced a single surviving daughter, with probability (1 − λ^*τ*^). The probability that an adult female tsetse dies after producing a single surviving daughter after surviving one pregnancy is thus:

$$p_{1,1}=\epsilon \lambda^{\nu +\tau }\beta \phi^{g}\left(1-\lambda^{\tau }\right).$$ (1)

Eq (1) can be generalized by induction to obtain the probability that a female tsetse produces *k* surviving female offspring after surviving *n* pregnancies. Thus

$$p_{n,k}=\epsilon \lambda^{\nu +\tau }\left(\begin{array}{c} n\\ k \end{array}\right)\beta^{n}\phi^{kg}{\left(\frac{1}{\beta }-\phi^{g}\right)}^{n-k},n>0;1\leq k\leq n,$$ (2)

where $\left(\begin{array}{c} n\\ k \end{array}\right)=\frac{n!}{(n-k)!k!}$ is the binomial coefficient.

Suppose *p*_0_, *p*_1_, *p*_2_, … are the probabilities that a female tsetse produces 0, 1, 2, … surviving female offspring in her lifetime, respectively. Suppose also that *p*_0_ + *p*_1_ < 1, to avoid the trivial case where a tsetse fly only produces 0 or 1 female offspring.

Summing Eq (2) over all *n*, gives *p*_*k*_, the probability that a female produces *k* surviving female offspring in its lifetime.

$$p_{k}=\frac{\epsilon \lambda^{\nu +\tau }\left(1-\lambda^{\tau }\right)\beta^{k}\phi^{kg}}{{\left(1-\beta \lambda^{\tau }\left(\frac{1}{\beta }-\phi^{g}\right)\right)}^{k+1}},k>0.$$ (3)

Eq (3) was used in [2] to obtain the mean and variance of the population size, extinction probability and time to extinction of populations of tsetse. Proofs of Eqs (1) and (2) are provided in [2] (Supplementary Information).

It can be shown easily that *p*_0_, *p*_1_, *p*_2_, … follow a geometric series, such that *p*_*k*_ = *bc*^*k*−1^, *k* = 1, 2, 3, …, where *b*, *c* > 0; and $p_{0}=1-\sum_{i=1}^{\infty }$. Eq (3) then becomes:

$$p_{k}=\frac{\epsilon \lambda^{\nu +\tau }\left(1-\lambda^{\tau }\right)\beta \phi^{g}}{{\left(1-\beta \lambda^{\tau }\left(\frac{1}{\beta }-\phi^{g}\right)\right)}^{2}}{\left(\frac{\beta \phi^{g}\lambda^{\tau }}{(1-\beta \lambda^{\tau }\left(\frac{1}{\beta }-\phi^{g}\right)}\right)}^{k-1},$$ (4)

where $b=\frac{\epsilon \lambda^{\nu +\tau }\left(1-\lambda^{\tau }\right)\beta \phi^{g}}{{\left(1-\beta \lambda^{\tau }\left(\frac{1}{\beta }-\phi^{g}\right)\right)}^{2}}$ and $c=\frac{\beta \phi^{g}\lambda^{\tau }}{(1-\beta \lambda^{\tau }\left(\frac{1}{\beta }-\phi^{g}\right)}$.

Following a framework developed by Harris [7], the generating function *g*(*θ*) of *p*_*k*_, is a fractional linear function given by;

$$g\left(\theta \right)=1-\frac{b}{\left(1-c\right)}+\frac{b\theta }{1-c\theta },0\leq \theta \leq 1.$$ (5)

### Extinction probability

The extinction probability for tsetse population is the non-negative fixed point of Eq (5), i.e. 0 ≤ *θ* ≤ 1 such that *g*(*θ*) = *θ*.

$$\theta =\frac{1-\lambda^{\tau }\left(1-\beta \phi^{g}\left(1-\epsilon \lambda^{\nu }\right)\right)}{\beta \phi^{g}\lambda^{\tau }},$$ (6)

where *βϕ*^*g*^λ^*τ*^ ≠ 0. In practice, 0 < *β* < 1, 0 < λ < 1 and 0 < *ϕ* < 1. In other words, the survival probabilities for both adult females and female pupa, and the probability that a pupa deposited is female are all in the open interval (0, 1). This allows us to avoid the trivial cases where *θ* = 0 or *θ* = 1. Eq (6) is the solution for the situation where the initial population consists of just a single female fly. For *N* flies in the pioneer population, and assuming that the survival and reproductive rates of all individual flies are independent, the extinction probability is *θ*^*N*^.

### Local sensitivity analysis of *θ*

In this section, we perform local sensitivity analysis, otherwise known as elasticity analysis, on the extinction probability for tsetse populations. Given that the extinction probability *θ*, depends differentiably on each input parameter, the normalized forward sensitivity (elasticity) index of *θ* with respect to all input parameters is:

$$\Pi_{\rho_{i}}^{\theta }=\frac{\rho_{i}}{\theta }\frac{\partial \theta }{\partial \rho_{i}},i=1,2,...,7,$$ (7)

where *ρ*_*i*_ is the set of all input parameters of the extinction probability. This method has been used extensively in the literature to determine the sensitivity of the reproduction number *R*_*o*_ of epidemiological models to model parameters [4, 5, 15]. When the initial population consists of *N* female tsetse, the extinction probability is *θ*^*N*^. The sensitivity indices of *θ*^*N*^ with respect to all input parameters is;

$$\Pi_{\rho_{i}}^{\theta^{N}}=\frac{\rho_{i}}{\theta^{N}}\frac{\partial \theta^{N}}{\partial \rho_{i}}=N\frac{\rho_{i}\theta^{N-1}}{\theta^{N}}\frac{\partial \theta }{\partial \rho_{i}}=N\frac{\rho_{i}}{\theta }\frac{\partial \theta }{\partial \rho_{i}}=N\Pi_{\rho_{i}}^{\theta }.$$ (8)

Notice that, when there are *N* female flies in the initial population, the sensitivity indices of *θ*^*N*^ for all input parameters is the sensitivity indices of *θ* multiplied by *N*. The larger the size of the initial population, the more sensitive extinction probability is to input parameters.

Writing Eq (6) in terms of the daily mortality rate for adult females (*ψ*), and the daily mortality rate for female pupae (*χ*), yields:

$$\theta =\frac{1-{\left(e^{-\psi }\right)}^{\tau }(1-\beta \;{\left(e^{-\chi }\right)}^{g}(1-\epsilon \;{\left(e^{-\psi }\right)}^{\nu }))}{{\left(e^{-\psi }\right)}^{\tau }{\left(e^{-\chi }\right)}^{g}\beta }.$$ (9)

Table 1 shows the derivations of the sensitivity indices of extinction probability with respect to all seven input parameters. These expressions were derived from Eqs (7) and (9) with a simple code in MAPLE 17 environment.

**Table 1:** Expressions for the sensitivity indices of extinction probability for each parameter.

Parameters	The sensitivity of extinction probability (*θ*) to input parameters
*β*: Probability deposited pupa is female	$\Pi_{\beta }^{\theta }=-\frac{-1+{\left(e^{-\psi }\right)}^{\tau }}{-1+{\left(e^{-\psi }\right)}^{\tau }-{\left(e^{-\psi }\right)}^{\tau }{\left(e^{-\chi }\right)}^{g}\beta +{\left(e^{-\psi }\right)}^{\tau +\nu }{\left(e^{-\chi }\right)}^{g}\beta \;\epsilon }.$
*χ*: Daily pupal mortality	$\Pi_{\chi }^{\theta }=\frac{\chi \;g(-1+{\left(e^{-\psi }\right)}^{\tau })}{-1+{\left(e^{-\psi }\right)}^{\tau }-{\left(e^{-\psi }\right)}^{\tau }{\left(e^{-\chi }\right)}^{g}\beta +{\left(e^{-\psi }\right)}^{\tau +\nu }{\left(e^{-\chi }\right)}^{g}\beta \;\epsilon }.$
*ϵ*: Probability of insemination	$\Pi_{\epsilon }^{\theta }=\frac{{\left(e^{-\psi }\right)}^{\tau +\nu }{\left(e^{-\chi }\right)}^{g}\beta \;\epsilon }{-1+{\left(e^{-\psi }\right)}^{\tau }-{\left(e^{-\psi }\right)}^{\tau }{\left(e^{-\chi }\right)}^{g}\beta +{\left(e^{-\psi }\right)}^{\tau +\nu }{\left(e^{-\chi }\right)}^{g}\beta \;\epsilon }.$
*g*: Pupal duration	$\Pi_{g}^{\theta }=\frac{\chi \;g(-1+{\left(e^{-\psi }\right)}^{\tau })}{-1+{\left(e^{-\psi }\right)}^{\tau }-{\left(e^{-\psi }\right)}^{\tau }{\left(e^{-\chi }\right)}^{g}\beta +{\left(e^{-\psi }\right)}^{\tau +\nu }{\left(e^{-\chi }\right)}^{g}\beta \;\epsilon }.$
*ψ*: Daily adult mortality	$\Pi_{\psi }^{\theta }=-\frac{\psi ({\left(e^{-\psi }\right)}^{\tau +\nu }{\left(e^{-\chi }\right)}^{g}\beta \;\epsilon \;\nu +\tau )}{-1+{\left(e^{-\psi }\right)}^{\tau }-{\left(e^{-\psi }\right)}^{\tau }{\left(e^{-\chi }\right)}^{g}\beta +{\left(e^{-\psi }\right)}^{\tau +\nu }{\left(e^{-\chi }\right)}^{g}\beta \;\epsilon }.$
*τ*: Inter-larval period	$\Pi_{\tau }^{\theta }=\frac{\tau \;\text{ln}(e^{-\psi })}{-1+{\left(e^{-\psi }\right)}^{\tau }-{\left(e^{-\psi }\right)}^{\tau }{\left(e^{-\chi }\right)}^{g}\beta +{\left(e^{-\psi }\right)}^{\tau +\nu }{\left(e^{-\chi }\right)}^{g}\beta \;\epsilon }.$
*ν*: Time from female emergence to first ovulation	$\Pi_{\nu }^{\theta }=\frac{\nu \;\beta \;{\left(e^{-\chi }\right)}^{g}{\left(e^{-\psi }\right)}^{\tau +\nu }\epsilon \;\text{ln}(e^{-\psi })}{-1+{\left(e^{-\psi }\right)}^{\tau }-{\left(e^{-\psi }\right)}^{\tau }{\left(e^{-\chi }\right)}^{g}\beta +{\left(e^{-\psi }\right)}^{\tau +\nu }{\left(e^{-\chi }\right)}^{g}\beta \;\epsilon }.$

## Results

Table 2 shows the sensitivity indices of extinction probability for each input parameter at different values of extinction probabilities. For instance, the sensitivity index of *θ* with respect to *ϵ* (probability female is inseminated by a fertile male) decreases by > 60% when *θ* (extinction probability) approaches 1. Thus, at *θ* = 0.419, a 10% decrease in *ϵ* yields a 22% increase in *θ*, whereas, at *θ* = 0.96, a 10% decrease in *ϵ* will only yield an 8.7% increase in *θ*.

**Table 2:** List and description of parameters affecting extinction probabilities for tsetse populations, and the sensitivity indices for these parameters, at two different values of extinction probability.

Parameters & descriptions	Baseline values	Sensitivity indices	
		*θ* = 0.419	*θ* = 0.960
Daily mortality rate for adult females (*ψ* = −ln(λ))	0.02-0.03 per-day [16]	+1.030	+1.080
Daily mortality rate for female pupae (*χ* = −ln(*ϕ*))	0.01-0.025 per-day [16]	+0.507	+0.374
Probability deposited pupa is female (*β*)	0.5 [6]	-0.836	-0.832
Probability female is inseminated by a fertile male (*ϵ*)	1 [6]	-2.220	-0.870
Inter-larval period (*τ*)	9 days [6]	+0.875	+0.929
Pupal duration (*g*)	27 days [6]	+0.507	+0.374
Time from adult female emergence to first ovulation (*ν*)	7 days [6]	+0.158	+0.154

### Varying sensitivity indices of *θ* for all input parameters as a function of *χ*

**Fig 1:**
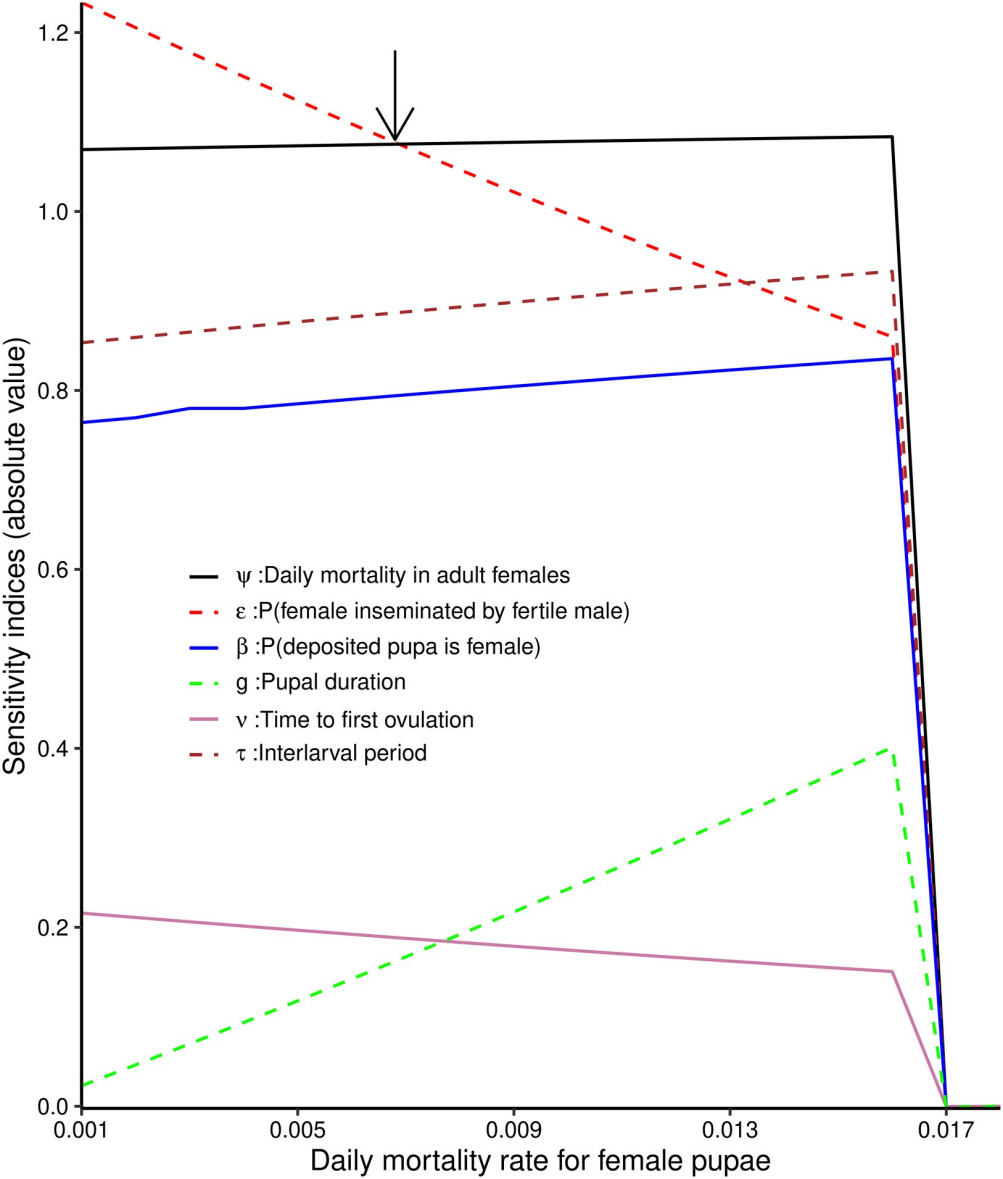
Variation in the sensitivity of extinction probability *θ* to six input parameters (*β*, *ϵ*, *ν*, *g*, *ψ*, *τ*) as a function of the values of the background daily rate (*χ*) of pupal female mortality. The sensitivity indices of extinction probability to six input parameters, in absolute value. The arrow through the plot indicates the point where *θ* becomes more sensitive to *ψ* than *ϵ*.

Here we investigate the changes that occur in the sensitivity indices of extinction probability for six input parameters as we vary *χ*, the daily mortality rate in female pupae. A simple script was written in MAPLE 17 environment to calculate the local sensitivity indices of *θ* with respect to the six remaining input parameters for different values of *χ*. Fig 1 shows changes in the sensitivity indices of *θ* with respect to each parameter as the daily mortality rate for female pupae (*χ*) varies from 0.1% to 2.5%, while keeping the other baseline values constant (Table 2).

As *χ* increases from 0.001 to 0.0065, the sensitivity index of *θ* with respect to *ϵ* reduces below the sensitivity index of *θ* with respect to *ψ*. At that point extinction probability becomes more sensitive to *ψ* than *ϵ*. When *χ* increases further to 0.013, the sensitivity of extinction probability to *ϵ* drops further below the sensitivity of extinction probability to *τ* (Fig 1).

### The performance of different control approaches when used in combination

**Fig 2:**
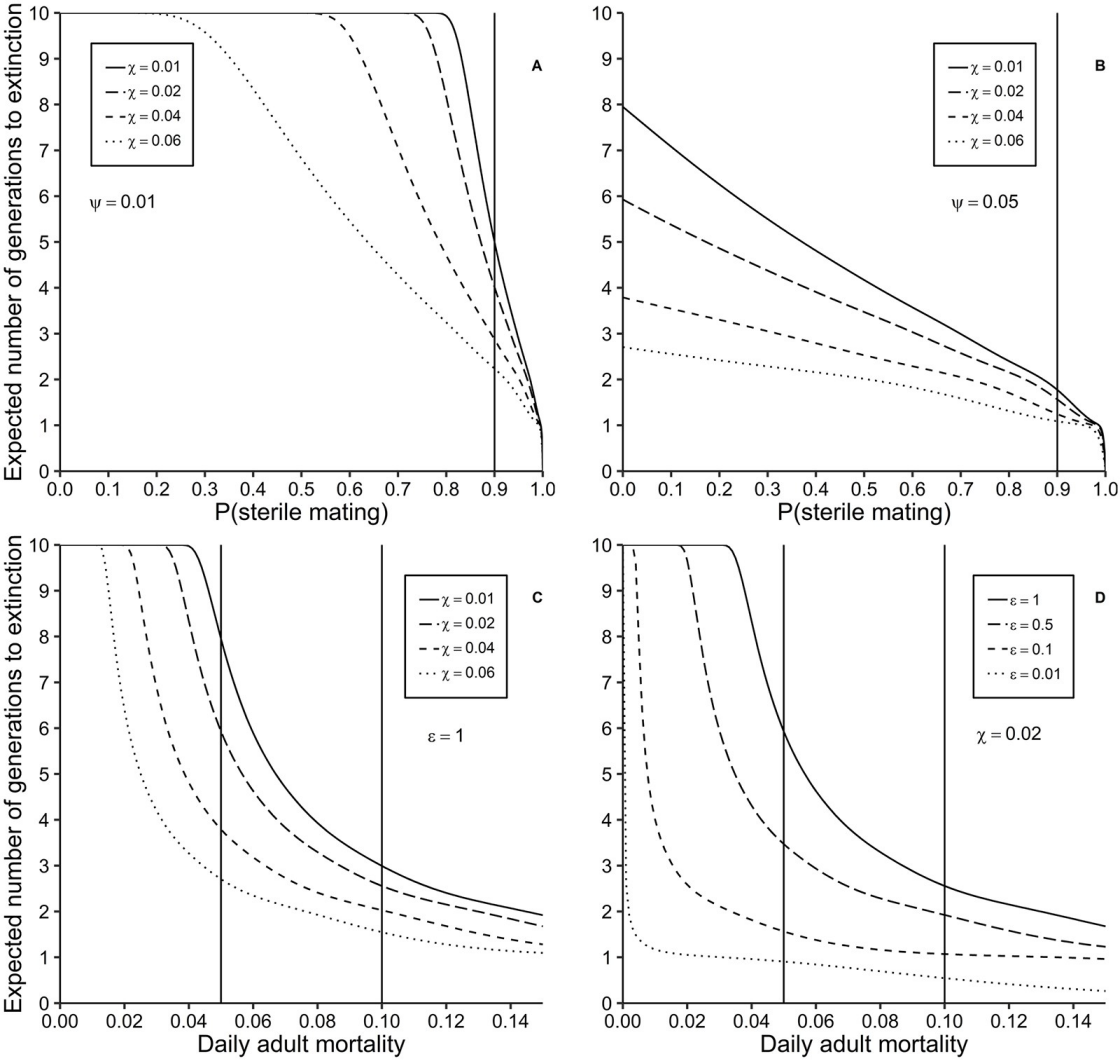
Expected number of generations to extinction as a function of various input parameters. Expected number of generations to extinction as a function of: A. The probability (1-*ϵ*) of sterile mating for different levels of daily pupal mortality (*χ*), at daily adult mortality *ψ* = 1%. B. The probability of sterile mating for different levels of daily pupal mortality, at daily adult mortality = 5%. C. Daily adult mortality for different levels of daily pupal mortality, and with no sterile mating: *ϵ* = 1. D. Daily adult mortality for different levels of probability of insemination, at daily pupal mortality = 2%.

The inter-dependence of sensitivity indices evident in Fig 1 suggests the need to consider the effects on the dynamics of a tsetse eradication campaign of using more than one control technique simultaneously. Accordingly, we estimated the times to extinction for various combinations of parameters affecting rates of mortality and reproduction. For example, the recently proposed Boosted SIT (BSIT) method would see sterile males treated also with the juvenile hormone analogue pyriproxyfen [17, 18], which would see simultaneous decreases in pupal production, and increases in the mortality of those pupae that are produced. As expected, for a given level of sterile mating, the time to extinction decreases as pupal mortality increases. This effect is also impacted by the background level of adult female mortality (*ψ*) (Fig 2A and 2B). For example, if 1 − *ϵ* = 0.9 and *ψ* = 1% per day, the expected times to extinction of a pioneer population of 1000 female tsetse are 4.9 and 2.2 generations when pupal mortalities are 1% per day, or 6% per day, respectively (Fig 2A). Adding the extra pupal mortality thus reduces the time to extinction by 2.7 generations, or 55.1%. The difference is much smaller, however, when *ψ* = 5% per day: now the times to extinction are 1.7 and 1.1 generations, respectively, for the above levels of pupal mortality (Fig 2B).

When the primary focus of the control is to kill adult females, as when using insecticide-treated targets for example, the advantage of killing pupae simultaneously is small when adult mortality is of the order of *ψ* = 10% per day—as is the case for the use of targets against *G. pallidipes* [19]. In that case the time to extinction is only 2.9 generations even with a background pupal mortality of *χ* = 1% per day (Fig 2C), declining only slightly to 1.5 generations if *χ* is increased to 6% per day. When *ψ* = 5% per day, the difference is greater: 7.9 and 2.7 generations for *χ* = 1% and 6%, respectively.

A similar picture emerges when adult killing is combined with the sterilisation of adult females (Fig 2D). When *ψ* = 10% per day, the expected time to extinction is only 2.6 generations—even without the release of any sterile males (*ϵ* = 1). If the population is flooded to the point where sterile males outnumber the wild males by 100:1 (*ϵ* = 0.01), 0.5 generations are required to achieve extinction, a difference of only 2.1 generations. When *ψ* = 5% per day the decrease is more substantial—from 5.9 to 0.9 generations as *ϵ* decreases from 1 to 0.01 (Fig 2D).

### Global uncertainty and sensitivity analysis of *θ*

As the above results indicate, local sensitivity analysis may not be robust enough to capture the influence of all input parameter values on the extinction probability since there are interdependencies between input parameters. Accordingly, we also carried out global uncertainty and sensitivity analysis of the extinction probability for tsetse population.

The exact values of the input parameters are not known in field situations, where many of these parameters depend on temperature and other climatic factors. It is therefore important to quantify the uncertainty involved in estimating the extinction probability (*θ*). To achieve this, and to establish the most important input parameters, we use LHS and PRCC methods for the global uncertainty and sensitivity analysis of the extinction probability. The method follows the approach of Samsuzzoha et al [4].

#### Uncertainty analysis

We aim to analyse the uncertainty involved in quantifying extinction probability (*θ*) based on the uncertainties associated with the input parameters. Accordingly, in order to investigate the sensitivity to this uncertainty we sample values from distributions of these parametes. We define prior probability distribution functions for each of the input parameters, based on the studies done on the life cycle of tsetse published in the literature [16, 20]. The probability distribution functions are given in Table 3, where *β*, *N* and *U* denote beta, normal and uniform distributions, respectively. The vast majority of studies on which the parameters are based were carried out on *G. m. morsitans* Westwood, but the limited information available from the literature suggests that there are relatively minor differences, between species, in the rates of pupal production and development [21]. Moreover, given that the qualitative aspects of the life history are identical for all species of *Glossina*, and that unit changes in pupal or adult mortality will affect various species in the same quantitative manner, we can be confident that the equations developed here will apply to all species. What will differ, in ways that we are not currently in a position to judge, is the quantitative effect of changes in climate, and other aspects of the environment, on the survival and reproductive rates in different species of *Glossina*. More work is necessary, particularly on forest and riverine species, to elucidate the relationship between environmental variables and rates of mortality and reproduction in tsetse.

**Table 3:** List of parameters and their prior probability distributions.

Parameters	Prior probability distribution
*ψ*	*β*(0.4, 12)
*χ*	*β*(0.3, 12)
*β*	*N*(0.5, 0.01)
*τ*	*N*(9, 0.747)
*g*	*N*(30, 1)
*ν*	*N*(8, 0.011)

**Fig 3:**
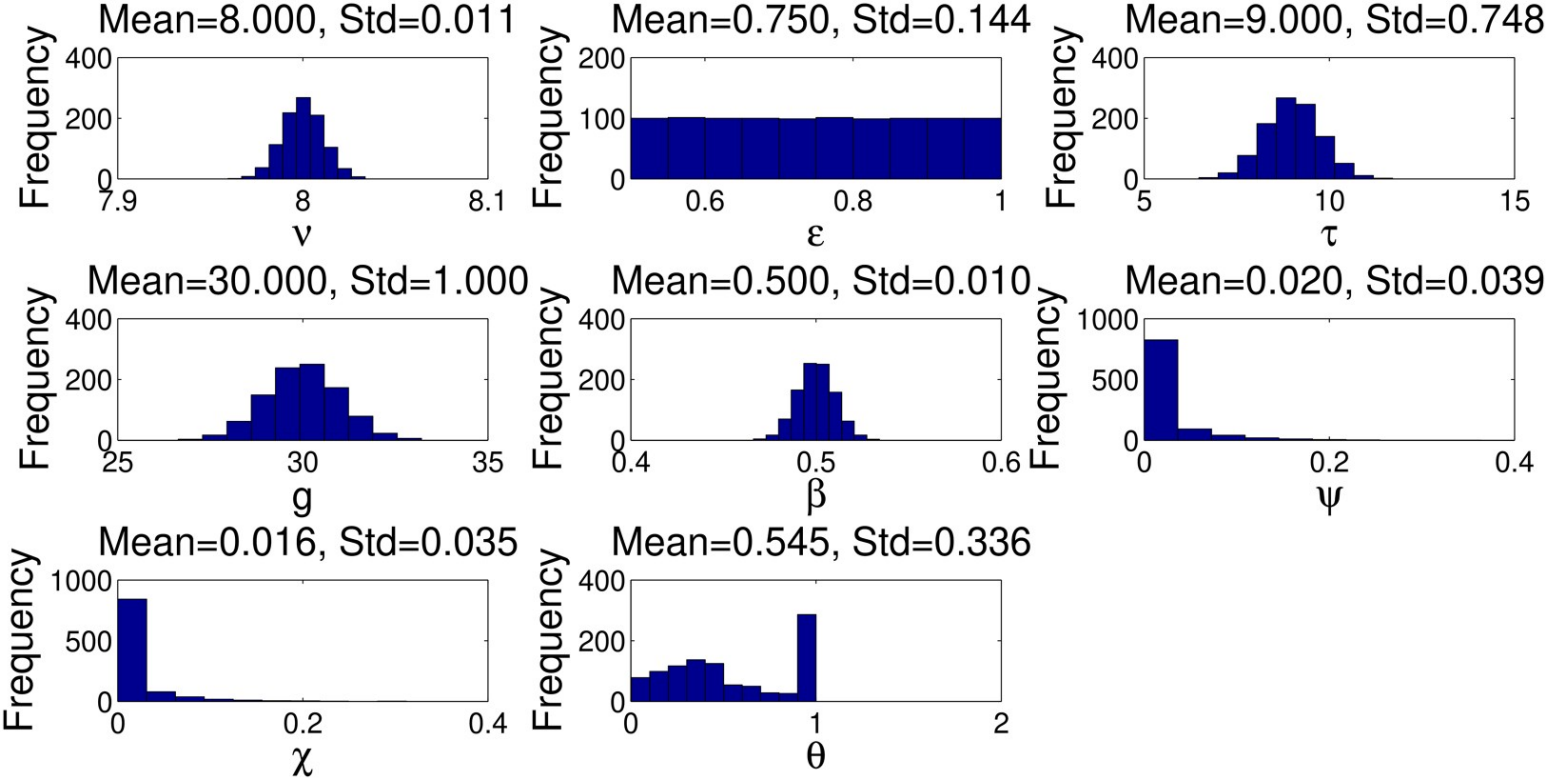
The uncertainty output for all input parameters, together with uncertainty output of the extinction probability, obtained from Latin hypercube sampling using a sample size of 1000 for the seven input parameters. (A). *β* is the probability deposited larva is female, *ϵ* probability female is inseminated by a fertile male, *g* the pupal duration and *ν* time from female emergence to first ovulation. (B). *τ* is the inter-larval period, *χ* the daily mortality rate for female pupae, *ψ* is the daily mortality rate for females and *θ* the extinction probability.

Using LHS, we obtain the uncertainty output for all the input parameters and also for the extinction probability. LHS is used to sample from the stratified probability distribution functions for different parameters, using 1000 intervals of equal probabilities. Fig 3 shows the uncertainty output for all the input parameters and the shape of their probability distribution, together with their summary statistics. The uncertainty output for extinction probability (*θ*) shows that it is beta distributed with mean = 0.545 and standard deviation = 0.336.

### PRCC/sensitivity indices of *θ* with respect to all input parameters

**Fig 4:**
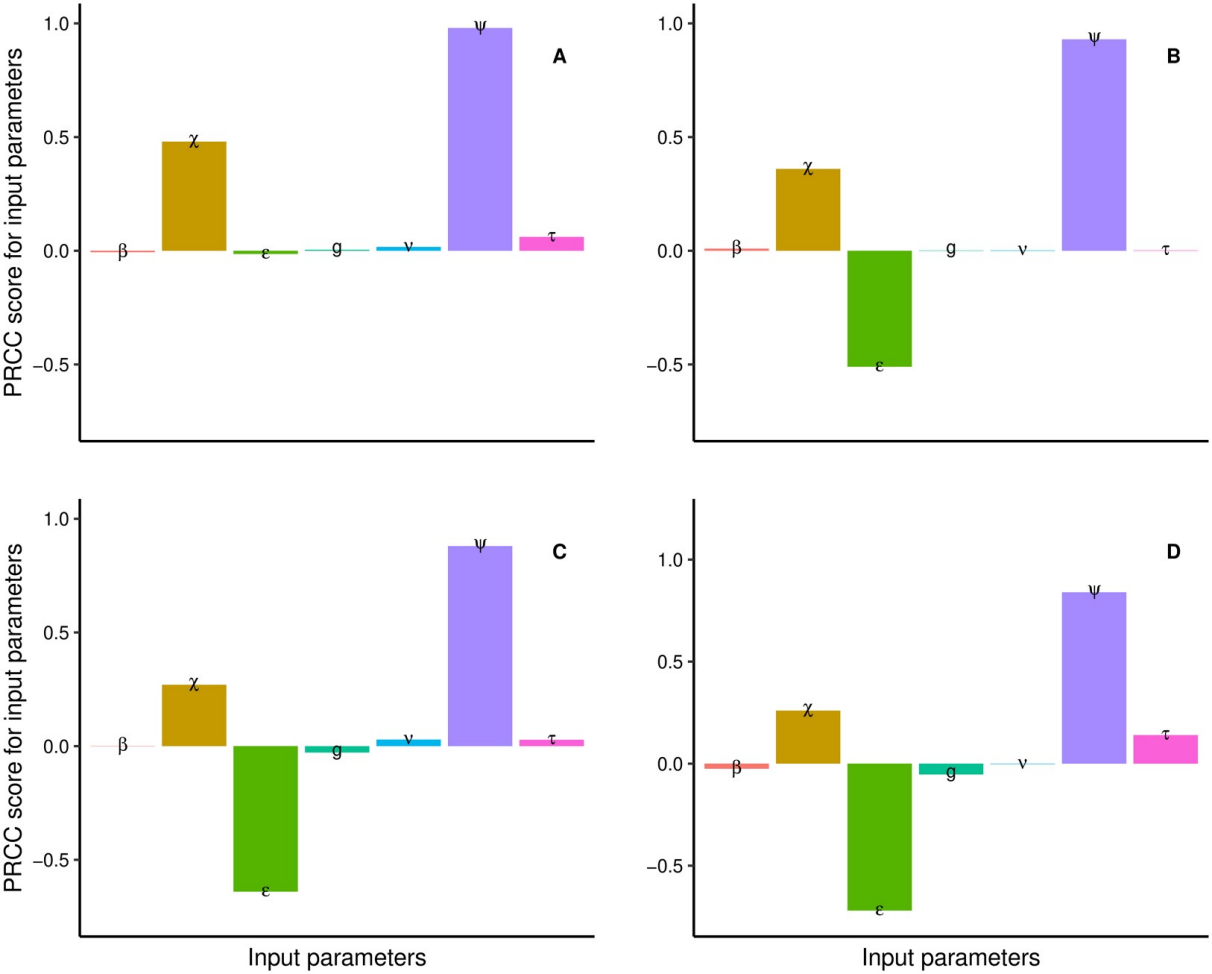
PRCC output for all input parameters with respect to the extinction probability. (A). Sampling *ϵ* between 0.999 and 1. (B). Sampling *ϵ* between 0.855 and 1. (C). Sampling *ϵ* between 0.51 and 1. (D). Sampling *ϵ* between 0.1 and 1. *ψ* is the daily mortality rate for females, *χ* the daily mortality rate for female pupae, *β* the probability deposited larva is female, *ϵ* probability female is inseminated by a fertile male, *τ* the inter-larval period, *g* pupal duration and *ν* time from female emergence to first ovulation.

To identify key input parameters, we carry out a sensitivity analysis by calculating the PRCC between each input parameter and the extinction probability. The parameter with the highest PRCC has the largest influence on the magnitude of the extinction probability. Fig 4 shows the PRCC outputs for all input parameters, where the probability (*ϵ*) that a female fly is inseminated by a fertile male is essentially equal to 1. In the field, males manage to find and mate with females, even at vey low population levels [22]. For most tsetse populations, the probability of insemination is thus close to 1. Accordingly, we allow *ϵ* to vary between 0.999-1. In Fig 4B, the prior probability distributions are kept the same, save for *ϵ* which is sampled between 0.885 and 1 [10, 11, 23].

LHS is used to sample from the prior probability distributions, where *ϵ* is sampled from a uniform distribution *U*(0.999, 1). Fig 4A shows that daily mortality rate for adult females (*ψ*) has a strong correlation with the extinction probability with PRCC score 0.91, followed by daily mortality rate for female pupae (*χ*) and inter-larval period (days) (*τ*), with PRCC scores of 0.47 and 0.058, respectively.

The female tsetse fly generally mates only once in her lifetime, storing the sperm in spermathecae and using small amounts to fertilize her eggs one at a time [24, 25]. When sterile males are introduced into a tsetse population, the probability (*ϵ*) that a female is inseminated by a fertile male falls below unity, by an amount that depends on the ratio of sterile to fertile males in the population.

The Sterile Insect Technique (SIT) has been used in attempts to control tsetse flies populations [26, 27] and was used to eradicate a small population of *G. austeni* on Unguja Island, Zanzibar, Tanzania [28]. The probability that a female is inseminated by a sterile male is 1 − *ϵ*. We allowed baseline values of *ϵ* to vary over a wide range, in order to assess the sensitivity of extinction probability to changes in *ϵ*, at varying baseline levels of the proportions of sterile males in the population. Fig 4 show the PRCC scores when *ϵ* is uniformly distributed either as U(0.855, 1), U(0.51, 1) or U(0.1, 1). The PRCC scores for *ϵ* in these three scenarios were -0.51, -0.64 and -0.72, respectively. Thus the absolute value of the PRCC score for *ϵ* increases as we allow more variability in the probability distribution function.

## Discussion

The simple life history of the tsetse fly enabled us to model its population dynamics as a stochastic branching process. We derived an expression for the extinction probability for tsetse populations and performed local and global sensitivity analyses, as well as global uncertainty analysis, on the extinction probability. We calculated all results for two fixed baseline values for *χ*, the pupal mortality rate, corresponding to values that resulted in low or high extinction probabilities. We obtained the sensitivity indices of the extinction probability to seven input parameters. When the extinction probability (*θ*) is fixed at either low or high levels (0.419 or 0.960) *θ* is more sensitive to changes in daily adult mortality (*ψ*) and the fertile insemination probability (*ϵ*) than to any of the other parameters. For a change in *θ* from 0.419 to 0.960, the sensitivity index of *θ* with respect to *ψ* increases by 0.05, whereas the change with respect to *ϵ* is larger, at 1.35 (decrease in absolute value) (Table 2). The parameters *ψ* and *ϵ* are important as they underpin the two main approaches to tsetse control. Hocking et al [29] broadly classified tsetse control and elimination techniques including game destruction, bush clearing, use of insecticides and biological control. These techniques can be pooled into two fundamental control philosophies—those which aim, primarily, to increase mortality rates in adult flies and those, like SIT, which aim to reduce tsetse birth rates [27]. Sensitivity analysis will indicate which parameter out of the two has the highest impact on the extinction probability.

From Table 2, observe that the sensitivity indices of *θ* to the input parameters depend on the value of the extinction probability. We allowed the daily mortality rate for pupae (*χ*) to vary from 0.001 to 0.025. The lower and upper bound values result in low and high extinction probabilities, respectively. We then calculated the sensitivity indices of *θ* with respect to the remaining six parameters. Fig 1 shows that the sensitivity of *θ* to each of the input parameters changes as extinction probability increases with increasing values of *χ*. Observe that for *χ* ≥ 0.018, the sensitivity indices of all the six parameters converged to zero. This is expected since the set baseline parameters values for all input parameters correspond to an extinction probability (*θ*) = 1 at *χ* ≥ 0.018. This can be verified easily, by substituting parameter values into Eq (6).

LHS and PRCC provide a suitable technique for assessing the impact of input parameters on the output and therefore inform possible choices for effective control efforts [30]. We defined prior probability density functions for the seven input parameters and we sampled from intervals of equal probability using LHS. The PRCC score of all input parameters was obtained for three sets of the probability distribution function, fixed for six parameters and varied only for *ϵ*. In all cases, *ψ* has the strongest impact on the extinction probability. The PRCC score for *ϵ* increases as we allow for more variability in its prior probability distribution.

The effectiveness of SIT is highly dependent on the daily mortality rates of female pupae. As the daily mortality for female pupae increases, extinction probability becomes less and less sensitive to the probability a female is inseminated by a fertile male (Fig 1). In contrast, the sensitivity of extinction probability to daily female mortality is almost constant, regardless of the daily mortality rates for female pupae. Previous theoretical studies and practical control campaigns have established the prime importance of increasing adult female mortality as a means of eradicating tsetse and trypanosomiasis [2, 31]. Our sensitivity analysis supports this conclusion and shows how the probability of extinction varies with small changes in adult mortality. A maximum daily birth rate of about 3% per day in tsetse means that as death rates—whether natural or imposed—exceed 3%, population numbers decline at an increasingly rapid pace. This will be true for all tsetse, whether savannah, riverine or forest species. What is less clear is the extent to which it is possible, for different species of tsetse, to impose the required increases in mortality.

A single insecticide-treated target baited with acetone and 1-octen-3-ol kills 0.5% and 2.5%, respectively, of adult female *G. m. morsitans* and *G. pallidipes* If used at a density of 4 targets per sq km this would result in mortalities of 2% and 10%, respectively [31]. The later identification of two attractive phenols and improved target design, led to an approximate doubling of target efficacy for the above species [32–34].

Riverine tsetse, such as *G. palpalis* and *G. fuscipes*, depend much less on odor for the detection of hosts. Smaller proportions of a population can therefore be attracted to an individual trap or target. These flies will, however, approach much smaller targets (7% of surface area of targets used for savannah tsetse) unaccompanied by odor. For *G. f. fuscipes* it is estimated that each of these so-called “tiny target” kills 0.2-0.3% per day of adult females [35]. The targets are so cheap that they can be produced in huge numbers, and so small that they can be easily and rapidly deployed. Used at an appropriate density they can thereby provide a sufficient increase in mortality among riverine tsetse that vector control could be an important component of the elimination of Gambian sleeping sickness [36, 37].

Similarly, insecticide treated cattle have been used to good effect in the control of trypanosomiasis in many situations, including those where riverine flies are the vectors, for example in controlling Rhodesian sleeping sickness in Uganda [38]. We may thus be confident that our sensitivity analyses support the idea that, for both savannah and riverine flies, it is possible to envisage increasing adult female mortality to the point where tsetse can be eliminated.

As illustrated in this study, and previously, tsetse population growth rate is also very sensitive to changes in the probability that an adult female is inseminated by a fertile male. This suggests alternative approaches to vector control, aimed at reducing the birth rate. In practice this currently involves SIT. It is, however, agreed that SIT cannot practically be used as a stand-alone technique to achieve tsetse eradication. Instead, it can be used against small remnant populations following major reductions in fly numbers achieved using insecticidal techniques [28]. In the event that eradication can be achieved using only the insecticidal technique then this will, of course, save the large extra cost due to an additional SIT operation. This situation arose, for example, in the elimination of *G. m. morsitans*, using odor-baited targets, from the Umfurudzi wildlife area in Zimbabwe [39] and of *G. m. centralis*, using aerial spraying, from the Okavango Delta of Botswana [40]. In neither case was it necessary or desirable to use SIT.

Laroche has recently suggested a new approach, termed Boosted SIT (BSIT), where sterile male tsetse are treated with the juvenile hormone analogue, pyriproxyfen, prior to release [17, 18]. Treating adult tsetse with pyriproxyfen does not affect their survival, but treated females produce larvae that die before they complete development. This has been demonstrated in the laboratory and in the field [41–44]. The idea behind BSIT is that the sterile males released would affect the wild tsetse population in two ways. First, virgin females with which they mate successfully do not produce any larvae. In terms of our model, *ϵ* decreases. Second, even if the male fails to mate with the female, transfer of pyriproxyfen to the female results in the death of any pupae produced. In terms of our model, *χ* increases. BSIT would thus result in simultaneous decreases in the probability of successful insemination, and increases in pupal mortality. Our sensitivity analysis shows, however, that as *χ* increases, there is a sharp decrease in the absolute value of the sensitivity index for *ϵ* (Fig 1). That is to say, increases in the efficacy of pyriproxyfen result in a decreased efficacy of SIT. From Fig 2 it is also clear that, in a situation where sterile males are released to the point where (1-*ϵ*) = 0.9, increasing adult female mortality (*ψ*) has a bigger impact on the time to extinction than increasing pupal mortality (*χ*). Thus, when *ψ* = 0.01 the time to extinction is 2.2 generations, even when *χ* is increased to 0.06. When *ψ* is increased to 0.05, however, the period is even shorter at 1.7 generations—even when pupal mortality *χ* is only 0.01. More careful analysis will thus be required to judge the extent to which the combined use of SIT and pyriproxyfen changes efficacy and cost effectiveness—and the relative merits of this approach and the simultaneous use of SIT and insecticide-treated targets.

Notice that the above problem does not occur for interventions where adult female tsetse are killed: increases in pupal mortality result only in a slight increase in the absolute value of the sensitivity index of *ψ*, the adult female mortality (Fig 1). There would thus be a simple additive effect of killing pupae as well as adult females. Nobody has, however, suggested a suitable means of combining these two killing methods. Using both insecticide and a juvenile hormone analogue, such as pyriproxyfen, on the same target makes little sense. As long as the insecticide is effective the presence of the pyriproxyfen would be irrelevant.

### Conclusions and limitations

In all scenarios considered in the global sensitvity analysis of extinction probability, control techniques which can achieve high mortality rates for adult female flies have the strongest impact on extinction probability. SIT, which can reduce reproductive rates, without increasing mortality, can also have a strong impact on extinction probability—but cannot be used as stand-alone method for achieving tsetse eradication. Where insecticidal approaches, such as aerial spraying, or insecticide-treated targets, or cattle, can be used by themselves to achieve eradication, this will save the extra expense of adding the SIT component.

The major limitation of our approach is that it is necessarily a simplification of real situations. For example, our modelling framework assumes that the population is unperturbed by movements between tsetse patches. In other words, the population is closed, such that, when tsetse populations are depleted, they cannot be replenished by invading tsetse from neighbouring patches. Finally, our work is based on the assumption that tsetse experience fixed environmental conditions throughout their life history. This assumption is not true in the wild, where tsetse experience daily and seasonal changes in various climatic effects. In future work we will estimate extinction probabilities for flies experiencing the variable climatic conditions typical of field situations.
